# Cultural differences in the relations between expressive flexibility and life satisfaction over time

**DOI:** 10.3389/fpsyg.2023.1204256

**Published:** 2023-08-02

**Authors:** Jen Ying Zhen Ang, William Tsai

**Affiliations:** Department of Applied Psychology, New York University, New York, NY, United States

**Keywords:** emotion, flexibility, life satisfaction, culture, expressivity

## Abstract

**Background:**

Expressive flexibility refers to the ability to assess situational demands and adjust one’s emotion expressions via enhancement or suppression. It has been associated with lower levels of depressive and anxiety symptoms and greater social acceptance. These relationships, however, have not yet been examined across cultures—where prior research has found cultural differences in norms on emotion displays and their associations with mental health. This study examined expressive flexibility across three cultural groups and their associations with life satisfaction and depressive symptoms over time.

**Methods:**

276 first-year college students (146 Asian American, 71 European Americans, and 62 Latinx Americans) completed two online surveys during the first (T1) and thirteenth week (T2) of the Fall 2020 academic semester.

**Results:**

Results revealed no significant cultural group differences in the ability to enhance or suppress emotions. However, we found a significant ethnicity x enhancement ability interaction in predicting T2 life satisfaction, controlling for T1 life satisfaction, age, gender, and emotion regulation frequency. Specifically, greater ability to enhance one’s emotions was significantly associated with higher life satisfaction over time among Asian Americans, but not for European Americans and Latinx Americans.

**Discussion:**

Our findings illustrate the importance of not looking just at cultural group differences in the levels of expressive flexibility, but also at the associations between expressive flexibility and mental health.

## Introduction

The ability to regulate emotions has been associated with psychological well-being and daily functioning. Recently, researchers have examined a facet of emotion regulation, expressive flexibility, which is the ability to *enhance* or *suppress* one’s emotional expressions according to situational demands (Bonanno et al., 2004; Westphal et al., 2010; Burton and Bonanno, 2016). For instance, in response to a friend’s good news, one may enhance displays of joy to demonstrate mutuality and understanding. In response to a boss’s mistake, individuals may suppress laughter to prevent further embarrassment. Higher levels of expressive flexibility has been associated with lower psychological distress, including lower levels of depressive, anxiety, and post-traumatic stress symptoms (Gupta and Bonanno, 2011; Shepherd and Wild, 2014; Rodin et al., 2017; Zhu and Bonanno, 2017; Chen et al., 2018; Levy-Gigi et al., 2020) and greater interpersonal functioning, such as lower levels of peer exclusion and greater likeability among peers and higher quality of relationships (Burton and Bonanno, 2016; Zhou et al., 2016; Wang et al., 2020). Expressive flexibility has also been associated with better adjustment to life stress, higher life satisfaction, and reduced burnout at work (Bonanno et al., 2004; Westphal et al., 2010; Chen et al., 2018; Lenzo et al., 2020).

Being flexible in enhancing and suppressing one’s emotional expressions requires that one is attuned to contextual cues and knowledge of social norms and expectations of the social situation. Because social norms and display rules about emotions are embedded within cultural systems that influences for that specific culture what is *socially appropriate*, the need for high levels of expressive flexibility and their subsequent associations with mental health may also vary across cultures. Previous research have shown that emotion display rules are associated with the type of emotions that are valued in each culture (Matsumoto et al., 2008). For example, individuals from individualistic cultures tend to focus on the independence and uniqueness of self (Markus and Kitayama, 1991), value high arousal positive states such as excitement more so than individuals from collectivistic cultures who value more calm states (Tsai et al., 2006). As such, they may be more motivated to upregulate or downregulate positive or negative emotions, respectively, after experiencing negative events (Miyamoto and Ma, 2011; Miyamoto et al., 2014). Individuals from collectivistic cultures focus on fitting in with the environment and its prevailing social norms and roles to maintain social harmony (Markus and Kitayama, 1991). As they value low-arousal positive states such as calmness more than individualistic cultures (Tsai et al., 2006), emotion suppression may be more culturally encouraged. Emotion suppression requires that one exercise control to repress one’s emotions, with elements of restraint, moderation and intensity reduction (King et al., 1992; Tsai and Lu, 2018). Consistent with these functions, studies have found that individuals from East Asian cultures display emotions that are more inhibited across a range of emotions and situations in daily life (Gross and John, 2003; Matsumoto et al., 2008; Tsai et al., 2016; Tsai, 2017; Cordaro et al., 2018; Tsai and Lu, 2018). Emotion suppression has been found to be used more frequently among individuals from East Asian cultures, and have mitigated associations with negative mental health outcomes (Butler et al., 2007; Cheung and Park, 2010; Soto et al., 2011; Tsai and Lu, 2018) compared to individuals from Western cultures. In contrast, individuals from Latinx American cultures, which are also considered collectivistic, provide an interesting contrast to European American and East Asian cultures, as they value expression of high arousal positive emotions but view negative emotions as less desirable (Ruby et al., 2012; Senft et al., 2022). Given that Latinx American cultures encourage expression of positive emotions, suppression of positive emotions have been found to be more detrimental to Latinx Americans compared to Asian Americans (Su et al., 2015).

These findings on cultural differences in emotion display converge to suggest that cultural scripts have key roles in influencing the extent to which people express or suppress their emotions, and their subsequent associations with mental health. To our knowledge, only one study thus far has directly examined cultural differences in expressive flexibility (Strickland and Skolnick, 2020), and found that the degree of one’s endorsement of collectivist values was associated with expressive flexibility levels. The authors also found that positive enhancement ability was negatively associated with trait anxiety for both United States and Indian samples, and that positive suppression ability and total flexibility levels were negatively associated with trait anxiety for United States but not Indian samples. Outside of that study, while a few researchers have investigated expressive flexibility among participants from other cultures (Bonanno et al., 2011; Chen et al., 2018, 2020; Fu et al., 2018; Lenzo et al., 2020), there has not been direct comparisons made across cultures. For instance, Chen et al. (2018) has found suppression ability to be associated with fewer symptoms of depression and anxiety and enhancement ability to be associated with higher life satisfaction in Chinese samples. It is reasonable to expect one’s ability to enhance or suppress emotion to similarly be shaped by the contextual norms and demands of an individual’s cultural background.

### Current study

The present study examined the levels of expressive flexibility among three cultural groups (i.e., European Americans, Asian Americans, and Latinx Americans) and their associations with psychological well-being over time. To examine the unique variance of expressive flexibility in psychological well-being, we explored if expressive flexibility predicted psychological well-being controlling for emotion regulation frequency (Chen et al., 2018). We hypothesized that European Americans, relative to Asian Americans, may be more apt at expressing and enhancing their emotions to communicate their inner dispositions to others and influence others (Kring et al., 1994; Kim and Sherman, 2007), and thus may have greater enhancement ability. Enhancement ability may be especially adaptive and more strongly associated with positive well-being outcomes for European Americans, relative to their Asian American counterparts. In contrast, Asian Americans may have greater suppression ability compared to European Americans, with greater ability to suppress their emotions given the emphasis placed on restraining emotions to maintain social harmony. Latinx Americans may endorse greater enhancement ability compared to Asian Americans and greater suppression ability compared to European Americans given the cultural emphasis on both positive emotion expression and on negative emotion suppression.

## Methods

### Participants and procedures

The present study involves secondary analysis of a larger IRB-approved study examining the impact of the COVID-19 pandemic among first-year college students. A total of 398 first-year college students from a large private university in New York were recruited through social media and emails on university listservs, and told that the purpose of the study was to understand first-year college students’ experiences during the COVID-19 pandemic. Participants completed baseline surveys on the first (T1) and thirteenth week (T2) of the Fall 2020 semester and were compensated with Amazon gift cards. At T2, 119 participants were lost to follow-up, though these participants did not differ in age and gender from those who completed the entire study. In total, data from 279 participants (M_age_ = 17.92, 70.1% female) were analyzed in the present study. 52.3% of them were Asian Americans (*n* = 146), 25.5% Latinx American (*n* = 71) and 22.2% European American (*n* = 62).

### Measures

#### Expressive flexibility

Expressive flexibility was assessed using the Flexible Regulation of Emotional Expression Scale (Burton and Bonanno, 2016). The 16-item scale consists of items such as “A friend wins an award for a sport that does not interest you,” or “Your friend is telling you about what a terrible day they had.” Participants rated how well they would be able to be more or less expressive than usual compared to how they were feeling on a Likert-scale, from 1 (“Unable”) to 6 (“Very able”). We examined overall expressive flexibility, as well as the Enhancement Ability and Suppression Ability subscales (ability to enhance or suppress emotion in a given situation). Construct validity was demonstrated with associations with measures of emotion regulation, personality and functioning, as well as performance on a laboratory task requiring participants to enhance or suppress emotion (Burton and Bonanno, 2016). Internal consistency of the current sample was good (Table 1).

**Table 1:** Bivariate correlations between expressive flexibility variables and well-being measures at T1 for Asian Americans/Latinx Americans/European Americans.

Study Variables	1.	2.	3.	4.	5.	6.	7.	8.	9.
1. T1 Expressive flexibility	--								
2. T1 Enhancement ability	0.78^*^/0.62^*^/0.68^*^	--							
3. T1 Suppression ability	0.78^*^/0.69^*^/0.82^*^	0.43^*^/0.12/0.35^*^	--						
4. T1 Reappraisal frequency	0.23^*^/0.21^*^/0.18	0.18^*^/0.09/0.21	0.23^*^/0.33^*^/0.10	--					
5. T1 Suppression frequency	0.13/−0.08/0.02	0.05/−0.21^*^/−0.17	0.19^*^/0.18/0.16	0.18^*^/−0.09/−0.05	--				
6. T1 Satisfaction with life	0.05/0.06/0.26^*^	0.13/0.10/0.28^*^	−0.08/0.07/0.07	0.06/0.26^*^/37^*^	−0.14^*^/−0.13/−0.23^*^	--			
7. T1 Depressive symptoms	−0.02/−0.10/−0.12	−0.02/−0.14/−0.03	−0.04/−0.10/−0.01	−0.17^*^/−0.35^*^/−0.39^*^	0.11/−0.03/0.32^*^	−0.41^*^/−0.44^*^/−0.52^*^	--		
8. T2 Satisfaction with life	0.15/0.08/0.14	0.21^*^/0.06/0.33^*^	0.04/0.06/−0.10	0.06/0.16/0.08	−0.30^*^/−0.24/−0.23	0.75^*^/0.72^*^/0.57^*^	−0.36^*^/−0.21/−0.07^*^	--	
9. T2 Depressive symptoms	−0.05/0.04/0.02	−0.05/−0.06/−0.20	−0.04/0.17/0.09	−0.03/−0.13/−0.04	0.28^*^/0.15/0.36^*^	−0.40^*^/−0.42^*^/−0.18	0.63^*^/0.46^*^/0.15	−0.53^*^/−0.48^*^/−0.49^*^	--
*α*	0.82/0.84/0.73	0.79/0.84/0.78	0.74/0.78/0.71	0.79/0.82/0.84	0.69/0.67/0.77	0.84/0.90/0.85	0.88/0.90/0.85	0.87/0.86/0.84	0.91/0.84/0.89

Correlations reported are for Asian Americans, Latinx Americans, and European Americans, respectively.

^*^p < 0.05.

#### Satisfaction with life

Satisfaction with Life was assessed using the Satisfaction with Life Scale (Diener et al., 1985). The five-item scale has items such as “In most ways my life is close to my ideal” or “The conditions of my life are excellent.” Participants rated their agreement with each statement on a Likert-scale from 1 (“Strongly disagree”) to 7 (“Strongly agree”). Construct validity was demonstrated through associations with measures of personality and well-being (Diener et al., 1985). Internal consistency of the current sample was good (Table 1).

### Depressive symptoms

Depressive symptoms were assessed using the Depression Anxiety Stress Scales-21 (Lovibond and Lovibond, 1995). The 21-item scale has three subscales measuring distress levels for depression, anxiety and stress. The depression subscale includes items such as “I felt that I had nothing to look forward to” and “I could not seem to experience any positive feeling at all.” Participants rated how much each statement applied to them over the past week, on a Likert-scale from 0 (“Did not apply to me at all – NEVER”) to 3 (“Applied to me very much, or most of the time—ALMOST ALWAYS”). These measures have good concurrent validity with other depressive and anxiety-related measures (Osman et al., 2012). Internal consistency of the current sample was good (Table 1).

### Emotion regulation frequency

Emotion regulation frequency was assessed using the Emotion Regulation Questionnaire (Gross and John, 2003). The 10-item scale has two subscales measuring the usage of cognitive reappraisal and expressive suppression, with items such as “When I want to feel more positive emotion, I change the way I’m thinking about the situation” or “When I am feeling positive emotions, I am careful not to express them” respectively. Participants rated their agreement with each statement on a Likert-scale from 1 (“Strongly disagree”) to 7 (“Strongly agree”). Construct validity of the scale was demonstrated with positive associations with measures of coping strategies and mood regulation. Internal consistency of the current sample was good (Table 1).

### Analytic plan

Analyses were conducted using Stata for Mac, v16.1. We first conducted descriptive statistics and bivariate correlations of the study variables, followed by one-way ANOVAs to examine whether expressive flexibility differed across cultural groups. Next, we conducted hierarchical regression analyses to examine prospective associations between T1 expressive flexibility and T2 well-being outcomes across ethnicities, controlling for T1 age, gender, well-being and emotion regulation frequency. Specifically, we entered T1 well-being variables, T1 age, T1 gender, T1 emotion regulation frequency and T1 expressive flexibility variables in Step 1, and entered the two-way interaction terms (T1 expressive flexibility variable x ethnicity) in Step 2.

## Results

Table 2 shows the descriptive statistics for expressive flexibility subscales across cultural groups. To test our first hypothesis of whether there were significant cultural group differences in expressive flexibility levels and its associated subscales, we conducted a series of one-way ANOVAs. Contrary to our hypothesis, there were no cultural group mean differences in all T1 expressive flexibility variables. Table 1 presents zero-order correlations of study variables. T1 expressive flexibility and enhancement ability was associated with T1 life satisfaction for European Americans. T1 enhancement ability was also associated with T2 life satisfaction for Asian Americans and European Americans. Interestingly, T1 suppression ability was not associated with life satisfaction. T1 expressive flexibility variables were not associated with T1 depressive symptoms.

**Table 2:** Descriptive statistics of expressive flexibility variables across cultural groups.

Variable	Asian Americans (*n* = 146; M_age_ = 17.9, 37.7% male)		Latinx Americans (*n* = 71; M_age_ = 17.9, 23.2% male)		European Americans (*n* = 62; M_age_ = 18.0, 31.6% male)		*F* (2,275)	*p*
	*M*	*SD*	*M*	*SD*	*M*	*SD*		
T1 Expressive flexibility	62.6	12.4	59.6	10.6	60.9	14.1	1.49	0.23
T1 Enhancement ability	34.4	6.5	34.9	6.6	35.3	7.1	0.46	0.63
T1 Suppression ability	33.5	6.8	31.2	5.6	32.7	7.9	2.66	0.07
T1 Reappraisal frequency	4.75	1.0	4.6	1.2	4.6	1.2	0.67	0.51
T1 Suppression frequency	4.0	1.1	3.6	1.3	4.0	1.2	2.65	0.07
T1 Satisfaction with life	21.1	6.6	23.0	7.3	22.0	7.0	1.87	0.16
T1 Depressive symptoms	5.9	4.6	5.4	3.7	6.6	6.6	1.25	0.29
T2 Satisfaction with life	21.4	7.1	23.4	6.4	22.0	6.3	2.13	0.12
T2 Depressive symptoms	6.6	5.4	6.5	4.8	7.5	5.1	0.91	0.40

^*^p < 0.05.

### Cultural group differences in prospective relations between T1 expressive flexibility subscales and T2 life satisfaction/depressive symptoms

**Figure 1:**
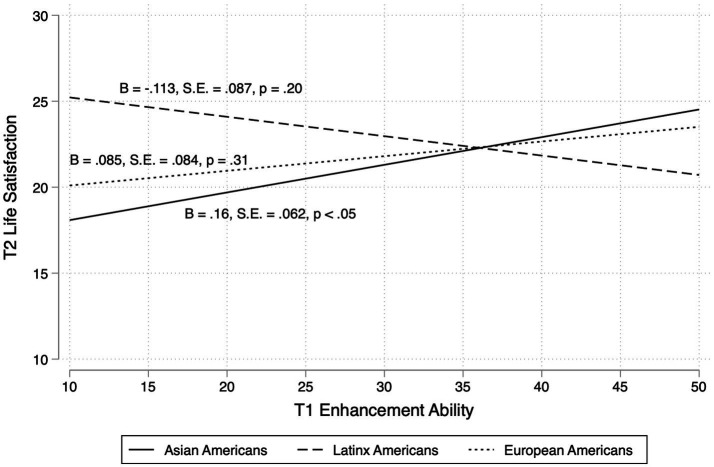
Prospective relations between T1 enhancement ability and T2 life satisfaction.

Controlling for age, gender, T1 life satisfaction, T1 emotion regulation frequency (i.e., emotion suppression and cognitive reappraisal usage), we found a significant ethnicity × enhancement ability interaction in predicting T2 life satisfaction [*F*(2, 265) = 3.35, *p* < 0.05]. Specifically, greater ability to enhance one’s emotions^1^ was associated with higher life satisfaction over time among Asian Americans (B = 0.16, SE = 0.062, *p* < 0.05), but this relationship was not significant for European Americans or Latinx Americans (Figure 1; Table 3). Interestingly, the relations between the ability to suppress emotions and life satisfaction was not moderated by cultural group. There were also no significant interaction effects between ethnicity × suppression ability or ethnicity × expressive flexibility in predicting life satisfaction. We also did not find cultural group differences in the relations between T1 expressive flexibility variables and T2 depressive symptoms.

**Table 3:** Hierarchical regression analyses of enhancement ability and life satisfaction.

*T2 Satisfaction with life*	B	SE	*R* ^2^	Δ*R*^2^	df	*F*
Step 1			0.52	--	(8,267)	36.44^***^
Gender	−0.62	0.64				
Age	−0.14	0.45				
^+^ Ethnicity (Latinx Americans)	0.39	0.70				
^+^ Ethnicity (European Americans)	0.18	0.73				
T1 Satisfaction with life	0.64^***^	0.04				
T1 Reappraisal usage	−0.10	0.27				
T1 Emotion suppression usage	−0.85^***^	0.25				
T1 Enhancement ability	0.07	0.045				
Step 2			0.53	0.012	(10,265)	30.34^***^
Gender	0.58	0.63				
Age	−0.09	0.45				
^+^ Ethnicity (Latinx Americans)	9.87^***^	3.3				
^+^ Ethnicity (European Americans)	2.77	3.70				
T1 Satisfaction with life	0.64^***^	0.04				
T1 Reappraisal usage	−0.11	0.27				
T1 Emotion suppression usage	−0.93^***^	0.25				
T1 Enhancement ability	0.16^***^	0.06				
^+^T1 Enhancement ability × Ethnicity (Latinx Americans)	−0.27^***^	0.11				
^+^T1 Enhancement ability × Ethnicity (European Americans)	−0.076	0.10				

^+^Ethnicity dummy coded with Asian Americans as reference category.

## Discussion

Our study is the first to our knowledge to examine expressive flexibility and their associations with psychological well-being among three cultural groups. Contrary to our expectations, we did not find the ability to enhance or suppress positive and negative emotions to differ across cultural groups. While this finding may appear at odds with prior research showing cultural differences in emotion display rules (Matsumoto et al., 2008) and the extent to which people expressed or suppressed their emotions (Su et al., 2015; Schouten et al., 2020; Tsai et al., 2021), our finding is corroborated by the study of Strickland and Skolnick (2020) which also did not find significant differences in expressive flexibility levels between United States and Indian samples. Moreover, the lack of cultural differences in the mean levels of expressive flexibility is consistent with a prior study among Asian American and European American college students that also failed to find cultural group differences in the extent to which they expressed their emotions. Another potential reason for the disparity in cultural findings between emotion regulation frequency (e.g., tendency to suppress emotions) and expressive flexibility is that expressive flexibility is conceptually distinct from emotion regulation frequency (Burton and Bonanno, 2016). It may be that one’s ability to enhance or suppress their emotion reflects their competency and control over its usage in appropriate settings, with a discernment on whether the emotion expression or suppression is aligned with the environmental or cultural norm. On the other hand, frequent usage of specific emotion regulation strategies may not be reflective of one’s ability to discern and calibrate the intensity and type of emotion display appropriate to a diverse range of settings, nor does it reflect the repertoire of other emotion regulation techniques that one may have in their toolbox. It is plausible that emotion regulation frequency indicates a conformity to the default coping style valued by the environment and culture rather than the ability to adapt to a range of settings and a deliberate, conscious assessment of the demands of the situation.

One primary aim of the current study was to understand whether the associations between expressive flexibility and psychological well-being differ across cultures. While prior work on expressive flexibility has found it to be associated with lower levels of depressive symptoms and higher life satisfaction (Gupta and Bonanno, 2011; Rodin et al., 2017), we found that overall expressive flexibility was not associated with life satisfaction or depressive symptoms over time for all three cultural groups. However, when examining enhancement and suppression ability separately, there appeared to be differences across this domain. Contrary to our hypothesis that relative to Asian Americans, enhancement ability would be more strongly associated with psychological well-being among European Americans, we found that greater enhancement ability was associated with higher life satisfaction over time among Asian Americans only. It may be that Asian Americans with greater enhancement ability reflect individuals who are more attuned to the social and relational norms of the dominant United States culture, and thus are more efficacious and effective in social settings in which emotions serve social functions. Greater abilities to enhance their emotional displays in social situations may in turn lead to improved interpersonal and intrapersonal outcomes. Moreover, East Asian Americans have been found to have greater attunement to relational harmony and context sensitivity in previous studies (Masuda et al., 2012; Imada et al., 2013; Ito et al., 2013), which may have also contributed to the well-being benefits of expressive flexibility for this cultural group. Finally, this finding was corroborated by a prior study among individuals from China, which also found a positive association between enhancement ability and life satisfaction (Chen et al., 2018). Nonetheless, more research is needed to better understand and replicate this cultural group difference. Contrary to the findings with enhancement ability, our results indicated that suppression ability was not associated with higher life satisfaction over time for all three cultural groups, despite well-known cultural differences in suppression usage and frequency (Su et al., 2015; Schouten et al., 2020; Tsai et al., 2021). This finding corroborates findings of Chen et al. (2018) in a Chinese sample, and also supports previous work suggesting that enhancement and suppression ability may have distinct influences on psychological well-being (Cameron and Overall, 2018; Chen et al., 2018; Strickland and Skolnick, 2020).

There are several limitations to our study that warrant consideration. First, our sample was limited to first-year college students in a large private university in New York at a time when the COVID-19 pandemic led to numerous social distancing restrictions (e.g., remote learning, discouragements of social activities, and absence of school-sanctioned social events). Mental health symptoms may also have been more elevated in this group given the disruption by the COVID-19 pandemic to their social and academic life in the midst of a crucial transitory period from high school to college. Given the social nature and function of emotion displays, it would be important for future studies to examine expressive flexibility and their associations outside of the pandemic period, or at a time when social distancing restrictions are lifted. Additionally, given findings of Strickland and Skolnick (2020) that one’s endorsement of collectivist values played a role in their expressive flexibility levels, and it is plausible that sample groups living in different geographic areas would have led to greater differentiation in their expressive flexibility levels, compared to our sample where all participants reside in the United States even as they are ethnically different. Third, while we focused on life satisfaction and depressive symptoms as indicators of psychological well-being, future studies can expand the scope of these measures by examining other important indicators, such as social functioning. Fourth, this study used self-reported ethnic group identification as a proxy for culture. Future studies can consider culture-specific or contextual dimensions (e.g., individualism–collectivism, emotion restraint or harmony values, and context sensitivity) to aid in our understanding of the source and mechanism behind the aforementioned cultural differences. Fifth, while we considered enhancement and suppression of positive and negative emotions collectively in this study, future studies can consider exploring greater nuances in the valence of emotions or type of emotions in enhancement or suppression ability. Finally, given the stability of life satisfaction and psychological well-being over 3 months, it may be that the short duration between the survey timepoints prevented an ideal test of the influence of expressive flexibility over time. Our recruitment also resulted in an unequal number of participants across the three ethnic groups. Future research with longer follow-up periods, more repeated measurements within individuals and with larger sample sizes are needed to advance our understanding of the mental health benefits of expressive flexibility.

This paper is the first to our knowledge to examine expressive flexibility across three cultural groups and their associations with psychological well-being over time. While our findings are preliminary and exploratory, we found that enhancement ability may be a culturally-unique indicator for well-being over time among Asian Americans, adding to findings within the nascent field of expressive flexibility. Future studies are needed to further investigate cultural differences in expressive flexibility and their associations with psychological well-being.

## Data availability statement

The original contributions presented in the study are included in the article/supplementary material, further inquiries can be directed to the corresponding author.

## Ethics statement

The studies involving human participants were reviewed and approved by NYU-IRB-FY2020-4261. The patients/participants provided their written informed consent to participate in this study.

## Author contributions

JA was the primary author on this manuscript and data analyses, while WT is JA’s PhD mentor supervising the project as well as leading the data collection process.

## Funding

The study was funded by the New York University Steinhardt Office of Faculty Development and Diversity—Diversity Innovation Grant.

## Conflict of interest

The authors declare that the research was conducted in the absence of any commercial or financial relationships that could be construed as a potential conflict of interest.

## Publisher’s note

All claims expressed in this article are solely those of the authors and do not necessarily represent those of their affiliated organizations, or those of the publisher, the editors and the reviewers. Any product that may be evaluated in this article, or claim that may be made by its manufacturer, is not guaranteed or endorsed by the publisher.

## Footnotes

^1^We conducted a post-hoc analysis to examine if this interaction finding was driven by the enhancement of negative emotions and/or positive emotions. We found that this interaction was primarily driven by the enhancement of positive emotions, but not negative emotions. Controlling for age, gender, T1 life satisfaction, T1 emotion regulation frequency, we found a significant ethnicity x positive enhancement ability interaction in predicting T2 life satisfaction. Specifically, greater ability to enhance one’s emotions positively was associated with higher life satisfaction over time among Asian Americans, but this relationship was not significant for European Americans or Latinx Americans.
